# Patient-reported outcome measures for clinical decision-making in outpatient follow-up: validity and reliability of a renal disease questionnaire

**DOI:** 10.1186/s41687-021-00384-0

**Published:** 2021-10-16

**Authors:** Birgith Engelst Grove, Liv Marit Valen Schougaard, Per Ramløv Ivarsen, Derek Kyte, Niels Henrik Hjollund, Annette de Thurah

**Affiliations:** 1 AmbuFlex, Center for Patient-Reported Outcomes, Regional Hospital West Jutland, Herning, Denmark; 2 Department of Clinical Medicine, Aarhus University, Aarhus, Denmark; 3 Department of Renal Medicine, Aarhus University Hospital, Aarhus N, Denmark; 4 School of Allied Health and Community, University of Worcester, Worcester, UK; 5 Aarhus University, Aarhus, Denmark; 6 AmbuFlex/WestChronic, Occupational Medicine, University Research Clinic, Aarhus University, Herning, Denmark; 7 Department of Clinical Epidemiology, Aarhus University Hospital, Aarhus N, Denmark; 8 Department of Rheumatology, Aarhus University Hospital, Aarhus N, Denmark

**Keywords:** Renal insufficiency, Ambulatory care, Patient reported outcome measures, Reproducibility of results

## Abstract

**Background:**

Patient-reported outcome measures are increasingly used by clinicians to support communication in telephone- or face-to-face consultations with patients. A renal disease questionnaire has been developed, but not sufficiently evaluated through clinimetrics in clinical setting. Hence, we aimed to evaluate the content validity, construct validity and the test–retest reliability of a renal disease questionnaire to be used for clinical decision-making.

**Methods:**

A content, construct validity and test–retest reliability study was conducted in 3 nephrology outpatient clinics in Central Denmark Region, Denmark. Content validity (face validity, comprehensibility and relevance) was assessed among 8 patients and 6 clinicians. Reliability was assessed by asking outpatients with chronic kidney disease to complete the questionnaire twice. Reliability was assessed by kappa statistics and agreement by percentage. Construct validity was determined using 4 a priori defined hypotheses and comparing 2 known groups.

**Results:**

Five new domains emerged, 6 items were rephrased and 3 items were removed following the content validity test. A total of 160 patients completed the questionnaire with median 8 days (IQR 2 days) between assessments. The test–retest reliability parameters of the single items in the questionnaire were substantial to almost perfect as all the observed weighted kappa values ranged from 0.61 to 0.91, 95% CI (0.34 to 0.95). In total, 61% of the single items showed almost perfect agreement. In total, 3 of the 4 hypotheses were accepted and 44% of the items showed satisfying known-group discriminative validity.

**Conclusion:**

A renal disease questionnaire used for clinical decision-making in outpatient follow-up showed acceptable content validity and substantial to almost perfect reliability. Sufficient construct validity was not established. Incorporating the questionnaire into routine clinical practice may improve the evaluation of disease burden in patients with chronic kidney disease.

**Plain English summary:**

We ask patients with chronic kidney disease (CKD) in Central Region Denmark to complete a questionnaire before each outpatient visit. The answers they provide are used to support communication with their health care provider. A questionnaire requires testing to ensure it can accurately capture important information about patient’s symptoms and quality of life. When questionnaires are used to support communication between patients and health care professionals, they need to have good measurement properties. This means they need to be: (1) trustworthy, (2) relevant to a patient’s health condition, (3) consistent and produce stable results every time. We explored the measurement properties of a questionnaire designed to be used in the face-to face outpatient visits for patients with CKD. We found that the questionnaire captured consistent and stable results. Using this questionnaire may help health care professionals to assess the patients´ burden of symptoms with a more patient-centered approach. Potentially, the use of the questionnaire will increase the patients´ ability to cope with their symptoms and strengthen patients´ involvement in the clinical decisions concerning their treatment.

**Supplementary Information:**

The online version contains supplementary material available at 10.1186/s41687-021-00384-0.

## Background

Chronic kidney disease (CKD) significantly impacts general health and well-being [1]. Patients with CKD are commonly frail, due to substantial comorbidity and significant symptom burden [1, 2]. The severity of CKD is categorized: CKD stages 1-3a are anticipated to be largely asymptomatic. However, in CKD stages 3b, 4 and 5, the symptom burden increases [3]. Common symptoms include fatigue, loss of appetite, pruritus, restless legs and cognitive dysfunction [2, 4, 5].

In Denmark, outpatient follow-up in patients with CKD stage 3b-5 has traditionally been based on regular face-to-face consultations. However, since 2012, there has been a growing interest in measuring and using patient reported outcomes (PROs) for remote data capture [6–8]. PRO measures capture information about a patient's health status directly from the patient [9]. PRO measures provide important information regarding the patients’ perspective on the degree and impact of disease symptoms [10]. Recent studies support the use of PRO measures in clinical practice with improved shared decision-making [11, 12], patient-clinician communication [13–16], promoting accuracy of symptom assessment [17], and patient self-management [18–20]. Remote PRO data may also help in managing use of healthcare resources [21–23]. Several PRO instruments used to monitor health status among patients with CKD have been evaluated [24] and previous studies have explored the implementation of PRO instruments in a pre-dialysis population [25–27]. The Kidney Disease Quality of Life-36 (KDQOL-36) questionnaire has been recommended in pre-dialysis patients, even though it was developed for patients in dialysis care [24, 28]. The KDQOL-36 has been validated in a Danish setting, but lacks evidence to support important properties on internal consistency, reliability and construct validity. Moreover, it is only tested in patients on dialysis in a Danish setting*.* [29]. Another study developed a clinical questionnaire [27], however this study was small and had several limitations such as low evidence for reliability and validity. Thus, there is a need for development of a reliable disease-specific instrument accurately measuring the most common symptoms, such as fatique, pruitus, nausea and loss of appetite, experienced by patients with CKD [1, 2].

In 2012, a renal disease questionnaire (RDQ) was developed and used to monitor general health status and to facilitate communication between patients and health professionals in the 3 nephrology outpatient clinics in Central Denmark Region with 858,083 inhabitants corresponding to 15% of the Danish population [30]. The RDQ was developed in close cooperation between clinicians and patients, and was tested for face-validity [31]. However, validity and reliability are essential psychometric properties for any measure [32, 33], and the ability of a PRO instrument to improve decision-making in clinical practice relies on the ability of the instrument to accurately capture the burden of disease or treatment [32]. The validity and reliability of the final questionnaire has not yet been evaluated, which is pivotal in the development of the instrument [32]. Therefore, we needed to demonstrate if the RDQ shows sufficient validity and is reliable so it may be deployed in clinical practice. This psychometric testing represents a first step toward supporting the questionnaire's use in clinical practice [34].

### Aims

We aimed to evaluate the face and content validity and test–retest reliability of the single items included in the questionnaire used as support in clinical decision-making in nephrology outpatient follow-up. Furthermore, we aimed to evaluate the construct validity of burden of symptoms by establishing known group validity.

## Methods

### Renal disease questionnaire (RDQ)

#### Historical development

The development of the questionnaire was iterative and based on consensus decision-making and face-to-face meetings with patients and clinicians. The process initiated in 2012 and was divided into two phases: (1) defining aim, content and construction of the questionnaire; and (2) pilot-testing. The renal disease questionnaire (RDQ) was implemented into clinical practice and was adapted based on experiences from clinical practice once yearly until 2017.

#### Content

The content was based on existing validated PRO instruments or items. Additionally, ad hoc items were developed if existing instruments or items were not available. This process was supported by nephrology specialists, a systematic literature search, and patient interviews. The prototype RDQ included information specific to aspects of daily life with CKD. The instrument consisted of 3 items regarding general health and fatigue from the Short Form-36 (SF-36) [35, 36]. SF-36 is a generic questionnaire with 8 subscales measuring physical and mental health [36], and the psychometric properties of the Danish SF-36 have been well documented [35, 37]. Seven items regarding lack of appetite, pruritus, dizziness, nausea, dyspnoea, reduced concentration and memory from the Kidney Disease Quality of Life Short Form (KDQOL-SF) [28] were included, some of which have been modified after the pilot test. The KDQOL-SF questionnaire combines the generic SF-36 scale and disease-specific components for assessing health-related quality of life in CKD patients [28]. Additionally, 5 items including frequent nocturnal urination, daily activities, worries about the future, oedema, and sleeping disorder were selected from the European Organization for Research and Treatment (EORTC) QLQ questionnaires [38] designed to measure quality of life in patients with cancer. However, these items are consistent with symptoms reported among patients with CKD [2, 5]. Finally, ad hoc items concerning e.g. drug adherence, blood pressure and weight were added.

#### Clinical use of the questionnaire

The questionnaire is used to support clinical decision-making and communication in nephrology outpatient clinics. A clinical expert group has assigned each item response into 3 colours: red, yellow or green. Red indicates that the patient is experiencing a particular problem within this domain; Yellow indicates that the patient is experiencing slight to moderate problems; and green indicates that the patient is not experiencing any problems within the domain. A graphical PRO overview of the patient response is embedded in the electronic health record [31].

### Study population

#### Face and content validity

In total, 8 patients participated in cognitive interviews and 6 clinicians participated in a focus group interview in order to evaluate face and content validity. Patients attending the nephrology outpatient clinics at Aarhus University Hospital and Central Regional Hospital, Viborg, were invited. Patients were selected purposively in order to maximise the variation of informants in terms of CKD duration, age, gender and experience of using the RDQ in the clinical encounter. Clinicians attending the focus group interview were selected based on their role in the organisation. Hence, participants from the management team, physicians and nurses were represented.

#### Construct validity and test–retest reliability

**Fig. 1:**
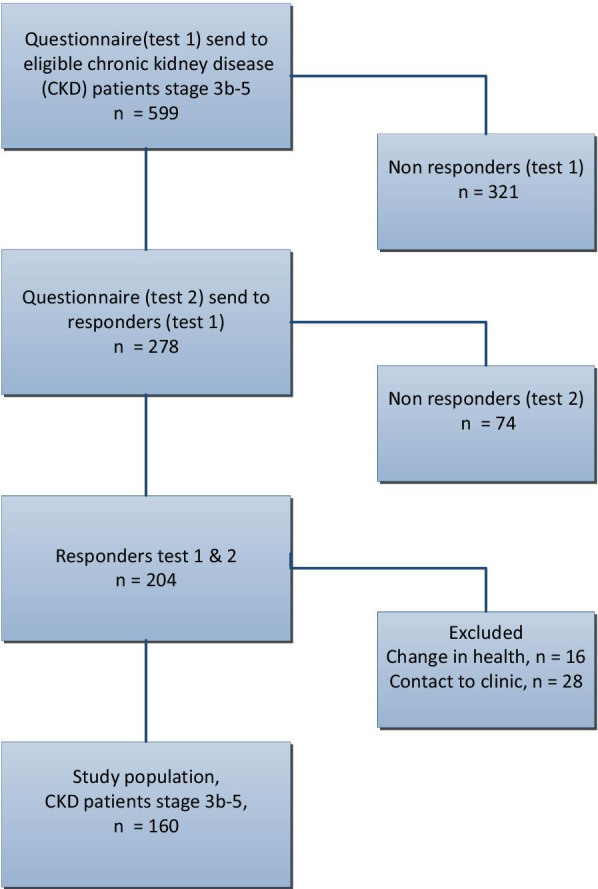
Inclusion of study participants

The target population was patients who had answered the questionnaire prior to a visit to the outpatient clinic within the previous 5 years. Inclusion criteria were age ≥ 18, CKD stage 3b-5 [3] in pre-dialysis care, as these patients often experience the symptoms asked in the questionnaire [2, 39–41]. All patients were contacted by “e-boks”, a secure electronic mailbox for all citizens in Denmark. The reliability analysis consisted of 160 responders of test 1 and test 2 and the construct validity analysis was based on the 278 responders of test 1. Figure 1 shows the inclusion of study participants.

### Procedure and analyses

#### Methods to evaluate content and face validity

Content validity includes face validity and reflects whether the items in a questionnaire represent the concept of interest [42]. The purpose of cognitive interviewing was to assess the patients’ and clinicians’ comprehension of the separate items and the complete PRO instrument against the intended meaning. Comprehensibility and usability, relevance and deficits of the questionnaire were assessed in one-to-one semi-structured interviews with 8 patients and 6 clinicians participating in a focus group. Table 1 shows the characteristics of the participants in the content and face validity analyses.

**Table 1:** Characteristic of the participants in the face and content validity study

	N (%)
Patients, semi-structured interviews (N = 8)	
Gender, men	6 (80)
Age, median [min;max]	72 [59;86]
Aarhus University Hospital	5 (62)
Central Regional Hospital, Viborg	3 (38)
Years followed-up in the outpatient clinic, median [min;max]	4 [1;8]
Clinicians, focus group (N = 6)	
Gender, men	1 (16)
Age, median [min;max]	51 [44;61]
Nurse	3 (50)
Physician	2 (33)
Team manager	1 (17)
Years of experience, median [min;max]	14 [6;27]
Aarhus University Hospital	6 (100)

Sampling followed the criteria of ‘sampling to redundancy’; that is to say interviewing people until no new information emerges [43]. Methodologically, the practice of cognitive interviewing patients as well as clinicians comprised 2 general techniques; “thinking aloud” and “verbal probing” while completing the questionnaires and also commenting on the layout and implication to clinical practice, following an interview protocol [44, 45]. All interviews were recorded and transcribed verbatim according to the interview guides shown in an Additional file 1. The researcher BEG, who is also a skilled interviewer and experienced nurse within the field of clinical nephrology, carried out the interviews and transcriptions.

#### Construct validity

Construct validity is defined by the COnsensus-based Standards for the selection of health status Measurement INstruments (COSMIN) panel as the degree to which the scores of a measurement instrument are consistent with hypotheses, e.g. with regard to internal relationships or differences between relevant groups [46]. According to the COSMIN criteria, the construct validity of an instrument is sufficient when 75% of the predefined hypotheses are confirmed in a sample of at least 50 patients [42]. We used the Wilson and Cleary conceptual model of health status as a theoretical framework to explain how constructs may be related and how symptom status affects the functional status and the patients´ general health perception. Each symptom represents a construct, which is a measure of a symptom [47]. In the present study, the construct of interest was the symptoms perceived by patients with CKD and the impairment of the physical function. Construct validity was assessed by comparing associations between selected single PRO measures and similar or divergent health-related outcomes.

Based on previous findings [7, 48, 49], we hypothesised that:

- The correlation between nocturnal awakening and low daily activity would be moderate to good (r =  > 0.50)
- The correlation between high self-rated health and low renal function would be negligible (r = 0.10–0.30)
- The correlation between lack of appetite and feeling aversion to food would be moderate to good (r = > 0.50)
- High Quality of life (EQ-5D) would correlate moderately to good (r = > 0.50) with high daily activities

We used Spearman rank correlation coefficients due to the ordinal nature of the items. Correlations were considered as follows: 0.10 to 0.30 = little or no relationship, 0.30 to 0.50 = fair relationship, 0.50 to 0.70 = moderate to good relationship, and > 0.70 = good to excellent relationship [50]. The shape and direction of the relationship between the selected items will be computed in Stata graph bars and reported. Finally, we assessed the capacity of each item in the questionnaire to discriminate between 3 subgroups of patients; patients in CKD stage 3b (n = 141), CKD stage 4 (n = 69) and CKD stage 5 (n = 23) [51]. Due to the low number of participants in stage 5, we combined participants in stage 4 and 5 in the analyses. We described the proportion and percentage of participants in each category and used the Mann–Whitney test for unequal distributions in these groups [46].

#### Reliability

Reproducibility was assessed by examining the degree of agreement between scores on the measure at first assessment and when reassessed [46]. The final version of the RDQ (Additional file 2) following the validity analyses formed the basis for conducting the test–retest analysis. Data collection took place from May to August 2019. Participants completed the questionnaire at 2 time points. By e-mail, patients were informed of the study details, and asked to complete the questionnaire (named ‘test 1’). Non-responders received a reminder after 2 days. Subsequently, the same questionnaire (named ‘test 2’) was sent to the responders of ‘test 1’. No reminders were sent. Time between ‘test 1’ and ‘test 2’ was 7 days. The test–retest study was performed in patients with stable disease activity, i.e. patients who in the test 2 questionnaire answered “somewhat the same” to the question: “Compared to a week ago, how is your health all in all now?” and “no” to the question: “During the past week have you been in contact with the outpatient clinic?”. Test–retest reliability and agreement were assessed within the item categories. In nominal and ordinal data, respectively unweighted and weighted kappa statistics with squared weights were used to assess reliability [32]. The 95% confidence intervals (CI) for weighted kappa values were measured using non-parametric bootstrap methods with 1000 replications [52]. The kappa agreement was interpreted as follows: < 0.2 (slight), 0.21–0.4 (fair), 0.41–0.60 (moderate), 0.61–0.80 (substantial), and 0.81–1.0 (almost perfect) [53]. Perfect agreement (identical responses at the two time points) and proportion of agreement was used to assess agreement measures [32]. A sensitivity analysis with patients who had been in contact with the outpatient clinic or who had reported a change in health condition was performed.

#### Other analyses

Descriptive statistics was presented as means and standard deviations (SDs), medians and interquartile ranges (IQR) or numbers (%) according to the distribution. Differences between responders and non-responders were evaluated by X^2^ test for categorical variables and the Mann–Whitney test for continuous variables with non-normal distributions. Lack of response was assessed for all items and was considered unacceptable if data was missing in more than 5% of an item category. Floor and ceiling effects were assessed and considered present if a high proportion (> 15%) of the respondents had a score at the lower or upper end of the scale [34]. We used the Charlson Comorbidity Index to estimate the burden of comorbidity [54, 55]. Information on the patients’ diagnosis and renal function was obtained from the Hospital Business Intelligence Register in Central Denmark Region [56].

A formal sample size calculation was not carried out in the present study due to the pragmatic design, where we approached all patients who had answered the questionnaire prior to a visit to the outpatient clinic within the previous 5 years. The overall recommendation regarding sample size in reliability studies is to include at least 50 patients [34]. A 5% significance level using two-sided tests was chosen, and STATA 16 was used for all analyses. This study followed the requirements from the COSMIN Risk of bias checklists for content validity, reliability and hypotheses testing for construct validity [57]. See checklist (Additional File 3).

## Results

### Content and face validity

The full results of the cognitive interviewing are shown in Additional File 4: Table S1. Three items in the questionnaire were removed, 6 questions were rephrased and 5 new items emerged. Clinicians reflected on the length of the questionnaire and relevance for the patients and underlined the importance of a short and concise questionnaire. Patients highlighted the importance of knowing the purpose of the questionnaire. The majority of patients found the content of the questionnaire relevant, and no critical comprehension difficulties were identified. The time used to fill in the questionnaire did not raise any criticism, average time for completion was 8 min (min 4; max 13). Upon revision, the final version of the RDQ (Additional file 2), provided the basis for psychometric analyses.

### Test–retest reliability and agreement of single items

In total, 599 questionnaires were sent out to patients at time point 1, and 278 questionnaires were sent out at time point 2. Questionnaire returns were 278 (46%) at time point 1 and 204 (73%) at time point 2, giving a total response rate of 34%. After exclusion of patients reporting a change in health (n = 16) and those who had been in contact with the outpatient clinic (n = 28), the study population consisted of 160 patients with CKD stage 3b-5 (Fig. 1). Patient characteristics are shown in Table 2.

**Table 2:** Patient characteristics measured at time for test 1 in responders and non-responders in test 2 among outpatients with chronic kidney disease (n = 555)

	Responders (n = 160) n (%)	Non-responders (n = 395) n (%)	*p* value
Gender, men	115 (71)	225 (57)	< 0.001
Age, year median [IQR]	69 [18.5]	56 [30]	0.04^b^
eGFR, median [IQR]	28.2 [10]	33,4 [10]	0.78^b^
CCI, mean (SD)	1.31 (1.5)	1,28 (1.31)	0.82
Low 0	49(30)	169 (42)	
Medium 1–2	88(55)	175 (44)	
High > 2	23(15)	51 (14)	
Quality of life (EQ-5D)^a^			
Median, [IQR]	0.80 [30]	0.80 [20]	0.97^b^
General health^a^			
Excellent	16 (10)	5 (7)	0.76^b^
Very good	37 (23)	22 (30)	
Good	68 (42)	30 (40)	
Fair	36 (23)	15 (20)	
Poor	3 (2)	2 (3)	

Patients reporting change in health and patients who have been in contact with the clinic are excluded

^a^Non-responders (n = 74) in test 2. CCI = Charlson Comorbidity Index; eGFR = estimated glomerular filtrations rate (renal function); EQ-5D = European Quality of life 5-Dimensions; IQR = Inter Quartile Range; *p*-value = Chi-squared or ^b^Mann-Whitney; SD = Standard Deviation

Non-responders were more likely to be younger (*p* = 0.04) and female (*p* < 0.001) (Table 2). The median age was 69 (IQR 18.5) years. In total, 67% reported suffering from other conditions, which affected their health besides CKD, such as arthritis, diabetes, pain, heart failure and cancer. The mean burden from comorbidity from the CCI was 1.31 (SD 1.5, range 0–8).

The median response time from test 1 to test 2 was 8 days (IQR 2, range 5–28 days). The test–retest reliability parameters of the single items in the RDQ were substantial to almost perfect as all the observed weighted kappa values were above 0.61, 95% CI (0.34 to 0.95). Perfect agreement ranged from 55 to 96% in each single item. Totally, 61% of all items showed almost perfect agreement, as shown in Additional file 5: Table S2. The sensitivity analysis including patients (n = 204) who had contact with the outpatient clinic or who reported a change in health condition showed slightly reduced to perfect agreement, ranging from 52 to 92% and kappa values between 0.56—0.87, 95% CI (0.37 to 0.92), as shown in Additional file 6: Table S3. Missing responses were less than 3% in all items. For the majority of items, a skewed distribution was observed with high proportions of more than 15% at the upper or lower end of the scale, as shown in Additional file 5: Table S2.

### Construct validity

Data supported the hypothesis in 3 of the 4 a priori constructed hypotheses. The results of hypotheses testing are shown in Table 3.

**Table 3:** Construct validity: correlations^a^ between selected PRO items among 278 outpatients with chronic kidney disease

	Daily activities	Renal function	Feeling aversion to food	Quality of life	Interpretation of correlation (*r*)	Shape of relationship
Nocturnal awakening	r = 0.31 *p* < 0.0001				Hypothesis rejected	Negative linear
Self-rated health		r = 0.19^b^ *p* = 0.004			Hypothesis accepted	Negative linear
Lack of appetite			r = 0.77 *p* < 0.0001		Hypothesis accepted	Negative linear
Daily activities				r = 0.58 *p* < 0.0001	Hypothesis accepted	Negative linear

^a^Spearman correlation coefficients (*r*) were calculated

^b^n = 233

The distribution of self-reported characteristics to show independence between 2 known groups determining the discriminative validity is presented in the Additional file 7: Table S4. In total 10 of the 23 items (44%) showed satisfying known-group validation in the RDQ.

## Discussion

This is the first study investigating some of the psychometric properties of a disease-specific renal disease questionnaire (RDQ) used to support clinical decision-making in outpatient clinics. Face and content validity was found acceptable. In total, 44% of the items showed satisfying known-group discriminative validity and 3 of the 4 a priori hypotheses were accepted, demonstrating the initial step of establishing construct validity. The test–retest reliability kappa values of the single items in the questionnaire were substantial to almost perfect. In total, 61% of the single items showed almost perfect agreement.

### Content and face validity

Patients emphasised the importance of knowing the purpose and clinical use of the questionnaire, which is crucial when implementing PRO measures in a clinical setting [11, 58]. Similar findings have been reported in previous studies [18, 19, 59, 60]. A recent systematic review found strong evidence supporting internal consistency and moderate evidence for construct validity for the KDQOL-36 in pre-dialysis patients [24], which formed the basis of the questionnaire in our study. The scale has been validated in a Danish setting, but has not yet reached recommended values [29]. The outpatient clinics needed an instrument to support clinical decision-making in terms of monitoring patients’ health status; this means focusing on the most relevant questions from a clinician- and patient viewpoint [15]. Consistent with previous studies [11, 61], our cognitive interviews supported that a questionnaire needs to address key issues and not be too comprehensive. The use of multiple ‘single-domain’ items within a measure allows the production of a short tool which, when compared to existing PROs, may be capable of measuring several patient-important domains using a less time-consuming format for patients and clinicians [62]. In contrast, single-domain items seem to lose sensitivity and are less reliable for tracking individual changes. However, the constructs measured in this questionnaire are unidimensional. Hence, a single item scale is considered appropriate [62]. Involving clinicians and patients in the evaluation of the questionnaire has potentially increased the feasibility and clinical relevance of the selected PRO measures.

### Construct validity

Evaluation of construct validity includes the degree to which a measure correlates with other measures to which it is similar and diverges from measures that are dissimilar [46]. Since no comparative instrument was available, we formulated hypotheses based on prior findings in the literature. The selected PRO measures in the analyses of construct validity were assumed to be the most clinically relevant when monitoring the progression of CKD [1]. With a 75% rate of confirmed predefined hypotheses in this study of 278 patients with CKD, according the COSMIN quality criteria we reached sufficient construct validity [42]. However, as each item in the questionnaire represents a unique construct and therefore needs to be validated with its own hypothesis, we cannot claim to have reached sufficient construct validity. However, construct validity is a lengthy, ongoing process [63, 64], and this paper only demonstrates the initial step towards establishing construct validity. In total, 10 of the 23 items (44%) in the RDQ showed satisfying known-group validation. Unfortunately, we needed to dichotomies data due to a low number of participants in the stage 5 group, and even though our findings are concurrent with the available literature [2, 7, 65], this may potentially have blurred the results and have violated the overall findings. Apparently, the symptom score varies widely in patients with the comparable renal function. Several studies suggest that also social, psychological determinants and comorbid conditions play an important role in symptom development and burden [66–68]. Generally, QoL deterioate when renal funcion declines [69]. However, the relation between kidney disease specific symptoms and decline in renal function is not straightforward and litterature on this is scarce [70]. In the EQUAL group, they found a faster decline in renal function to be associated with higher symptom burden [66]. This may play a role when known group validation is conducted, whilst we cannot rule out some adaptation to long-term CKD. During the development process, redundant items should have been added to the questionnaire for validation purposes [63]. Unfortunately, this was not feasible in the design of this study. It should therefore be acknowledged that further research is required to provide additional evidence around construct validity. Hence, the four a priori chosen hypotheses from the selected items do not reflect on the construct validity of items not included in the hypotheses. However, this questionnaire serves as a clinimetric scale and does not necessarily need to satisfy the same requirements as a psychometric scale [63]. From a clinimetric perspective, a single-item measure could be useful as long as it discriminates between different groups of patients and reflects clinically relevant changes over time [71]. However, further psychometric analysis investigating the validity of single items will be needed before full construct validity is established.

### Reliability

Overall, we found that test–retest reliability was considered substantial to almost perfect in all the included items in the RDQ, although some items showed only fair or moderate values at the lower end of the confidence intervals. However, kappa values are influenced by skewed distribution, number of classes, and systematic differences between the 2 measurements [72]. A skewed distribution leads to a lower extent of real agreement due to a higher fraction of chance agreement [72]. A skewed distribution in several items in the questionnaire was shown, probably due to a homogeneous population with stable disease activity and low symptom burden. This skewed distribution may have resulted in an underestimation of the kappa values [72]. A skewed distribution was observed with high proportions of more than 15% at the upper or lower end of the scale. Potentially, this could affect the reliability and the ability to distinguish patients with the lowest or highest score from each other [42]. From a clinician viewpoint this was considered acceptable as the items in the questionnaire represent alert symptoms, which indicate a potential hazard or condition requiring special attention. We found the lowest kappa values in the items concerning medication adherence 0.61 (95% 0.34; 0.83) and vomiting showing kappa value at 0.69 (95% 0.32; 0.90). However, the agreement in both items was high; > 98%. The discrepancy between level of agreement and kappa may be caused by an unequal distribution and ceiling effect. This illustrates that kappa is affected by the prevalence of the measured event and distribution of item scores [72]. The outliers with low agreement represented items as fatique (55% perfect agreement) and restless legs (62% perfect agreement), which may be related to a certain day to day variation. Several potential threats related to the consistency of a PRO measurement may occur. A long interval increases the risk of a real change in patient status and a short interval increases the risk of recall bias [63]. The study population consisted of frail elderly people with a high level of comorbidity; this required that the interval between the assessments should be short due to variation in health. We had a time difference at 8 days (IQR 2 days) between test 1 and test 2, which is within the recommendation from the literature [32, 34]. A real change in the patient´s health status between the 2 time points of measures might cause a potential error related to the consistency of a PRO measurement [63]. However, we excluded patients who reported a change in health status which strengthened our results. According to COSMIN framework, reliability needs to be tested in patients who are stable in the interim period on the construct to be measured [34]. Potentially, bias would occur if the patients failed to report a change in disease status due to recall problems. Another strength in the analyses was that the questionnaires were completed independent of a visit in the clinic and patients who had a visit at the outpatient clinic were excluded from the analyses (Fig. 1). If the disease status had changed upon treatment at the outpatient clinic, it might have induced risk of bias, as this represents responsiveness [34]. We performed sensitivity analysis including patients with a change in health condition or having contact with the outpatient clinic and found a tendency towards decreased reliability as expected.

A limitation in this study was the risk of selection bias. The response rate was only 34%, which might have been caused by the pragmatic design. This only allowed us to approach a known group of patients without any a priori knowledge of their present renal function. Compared to non-responders, there were more elderly and more men among responders, whereas no significant difference in renal function was observed. A study population that does not represent the targeted population may challenge generalisation of the results. However, the differences between responders and non-responders are not likely to be clinically important. Importantly, the presence of some selection bias cannot be excluded, thus hampering the external validity of our findings. A use of reminders at test 2 could have increased the overall response rate. However, the participants did not receive reminders at test 2 to ensure an acceptable interval length between the 2 measurement points in a test–retest study [42]. The mode of administration only included web responders, which could potentially induce selection bias. However, in recent years, the use of web-based questionnaires has increased dramatically [73]. This could potentially underestimate the reliability parameters due to the homogeneous study population. Yet, the study population represents the target population and the real-life environment and a mere of 5% of the patients in the outpatient nephrology clinics responded to the questionnaire in a paper version.

Among the 3 main measurement properties domains in the COSMIN framework, we have investigated the validity and the reliability. The present study was not designed to evaluate responsiveness, which should be assessed in a longitudinal study. This would provide further evidence when using the RDQ to follow patients’ health status over time.

Incorporating this questionnaire into clinical practice may allow measurement of outcomes that may be more relevant to approximately 600–800 patients with CKD using this questionnaire. In the coming years we expect a more intense use of PRO measures in clinical practice [73], which highlights the importance of improving the quality of this PRO instrument.

## Conclusion

This is the first study investigating some of the psychometric properties of a disease-specific renal disease questionnaire (RDQ) used to support clinical decision-making in outpatient clinics. Our study shows the importance of including end users when developing a questionnaire for clinical use. It is fundamental that the questions asked reflect the needs of the clinicians and patients. We have demonstrated the initial step in establishing construct validity. However, the construct validity and discriminative ability should be further investigated in future studies. Overall, RDQ showed substantial to almost perfect test–retest reliability. Further psycometric measurement properties and establishment of clinical responsiveness represents the final necessary steps before deploying this tool for use in clinical nephrology settings.

## Supplementary Information

**Additional file 1.** Interviewguide.

**Additional file 2.** Renal Disease Questionaire (RDQ).

**Additional file 3.** COSMIN Risk of BIAS checklist.

**Additional file 4. S1.** Results from the cognitive interviewing.

**Additional file 5.** Agreement and reliability between the items from test 1 to test 2 in original categories (n = 160).

**Additional file 6.** Sensitivity analyses showing agreement and reliability between the items from test 1 to test 2 in original categories (n = 204).

**Additional file 7.** Known group (discriminiative validity) stratified into CKD stage 3b and stage 4 and 5 at time for test 1 among 160 patients with chronic kidney disease.

## Data Availability

A non-identifier version of the dataset used in this current study is available. Interested researchers may contact the corresponding author for further guidance.

## Abbreviations

CCI: Charlson Comorbidity Index
CI: Confidence Intervals
CKD: Chronic kidney disease
COSMIN: COnsensus-based Standards for the selection of health status Measurement Instruments
EGFR: Estimated Glomerular Filtration Rate
EORTC: European Organization for Research and Treatment
EQ-5D: European Quality of life 5-Dimensions
IQR: Interquartile range
KDQOL-36: The Kidney Disease Quality of Life-36
PRO: Patient-reported outcome
RDQ: Renal Disease Questionnaire
SDs: Standard Deviations
SF-36: Short Form-36
