# Supplementary material for: Patient-reported outcome measures for clinical decision-making in outpatient follow-up: validity and reliability of a renal disease questionnaire
Source: J Patient Rep Outcomes. 2021 Oct 16;5:107. doi: 10.1186/s41687-021-00384-0 (PMC8520563; doi:10.1186/s41687-021-00384-0)
Supplement: Supplementary file 1 — Additional file 1. Interviewguide. [file 41687_2021_384_MOESM1_ESM.pdf]

Interview guide	
No, date:	
Sex:	
Age:	
Number of years followed in the outpatient clinic:	
Introduction, purpose and duration of the interview	
	In front of you is a questionnaire and I will ask you to complete this while you think aloud. Afterwards, I have a few overall questions relating to the questionnaire.
Questions asked while the patient is completing the questionnaire	
**Comprehensibility**	• After reading the introduction, to what extent do you feel confident in your ability to complete the questionnaire?<br>• What does this (word / phrase) mean to you?<br>• Could you repeat this, putting the question into your own words?<br>• I noticed you hesitated. Do you want to tell me your thoughts?<br>• Do you think it will be difficult for others to answer that question?
**Response options**	• Was it difficult to answer this question? Why?<br>• Is your answer among these possible response options?<br>• Could you tell me how you came up with this response (answer option) in this question? How did you decide?
**Topics**	• Could you explain what you mean by e.g. "an exhausting fatigue" in your own words?<br>• Where should we insert this question to make the most sense for you?
Follow-up questions (semi-structured interview)	
**Comprehensibility**	• To what extent do you feel confident in completing the questionnaire?<br>• Did you find some of the questions confusing or were some of the

	questions difficult to understand?<br>Were there any of the answer options you did not understand / or did not seem to make sense? Which? Why?
**Relevance**	• Did you find any topics in the questionnaire that you do not asso-ciate with your illness?<br>• Could we possibly remove any questions? Why? Repetitions?<br>• Which topics do you think are the most important in order to get the best possible description of how you feel on the day you an-swer this questionnaire?
**Comprehensiveness/Gaps**	• Do you find any issues or concerns relating to your illness missing in the questionnaire? Which?<br>• Could you think of one or more specific questions that could con-tribute to a better understanding of your illness? Which?<br>• To what extent do you feel that these questions help you to put your well-being into words?<br>• What do you think about the length of the questionnaire?
**Additions**	• Could you name something you would like to have been asked about, which is not present in the questionnaire?<br>• From what we have discussed here, do you have some additional comments or important summary points?

Interview protocol – focus group	
No:	
Sex:	
Age:	
Role in organisation:	
Introduction, purpose and duration of the interview	
	In front of you is a questionnaire and I will ask you to complete this while you all think aloud. During the process, I will ask you additional overall questions.
Questions asked while the clinicians complete the questionnaire (semi-structured interview)	
**Comprehensibility**	• After reading the introduction, to what extent do you feel confident in your ability to complete the questionnaire?<br>• Did you find some of the questions confusing or were some of the questions difficult to understand?<br>• Are there any of the answer options you did not understand / or did not seem to make sense? Which? Why?<br>• To what extent do you feel confident in completing the questionnaire?
**Relevance**	• In which situations do you experience that kidney disease affects patients the most? Why?<br>• Which questions are most important to you in the consultations?<br>• In your experience, which symptoms/questions indicate a worsening in the patients´ kidney function/or the patients´ well-being?<br>• In your opinion, which of these questions are the best indicators of the patients´ well-being?<br>• In your experience, does the questionnaire contain questions not relevant for patients to answer due to their illness? Which?<br>• Could we possibly remove any questions? Why? Repetitions?
**Comprehensiveness/Gaps**	• Do you find any domains relating to chronic kidney disease missing in the questionnaire? Which?

	• To what extent do you feel that these questions help patients to put their symptoms into words?<br>• What do you think about the length of the questionnaire?
Additions	• Could you think of one or more specific questions that could contribute to a better understanding of chronic kidney disease? Which?<br>• After our discussion, do you have some additional comments or important summary points?
