# Supplementary material for: Patient-reported outcome measures for clinical decision-making in outpatient follow-up: validity and reliability of a renal disease questionnaire
Source: J Patient Rep Outcomes. 2021 Oct 16;5:107. doi: 10.1186/s41687-021-00384-0 (PMC8520563; doi:10.1186/s41687-021-00384-0)
Supplement: Supplementary file 2 — Additional file 2. Renal Disease Questionaire (RDQ). [file 41687_2021_384_MOESM2_ESM.pdf]

QUESTIONNAIRE ON YOUR HEALTH DATA FOR OUTPATIENT USE

In this questionnaire, you must answer questions about your blood pressure, heart rate and weight. Please follow the instructions below.

Location of the cuff

- Blood pressure is measured on the same arm each time
- The cuff is placed around the bare arm and stretched around the upper arm, there must be no clothing tightening on the arm
- The cuff must not be tight. A finger must easily be placed between the cuff and the upper arm
- The cuff is placed on the upper arm a few centimetres above the elbow with the air tube to the middle and inwards

The measurement

- You should preferably measure your blood pressure in the morning before taking your morning medication
- You must sit comfortably reclined against the backrest in a chair WITHOUT crossed legs for 5 minutes and have the opportunity to sit undisturbed. Do not talk or move during the measurement
- You make 3 measurements at 2-minute intervals
- You must enter the last blood pressure you measured in the questionnaire

What is your blood pressure today?

Write the systolic blood pressure here: ___

Write the diastolic blood pressure here: ___

What is your heart rate today?

Write the answer here: ___

What is your weight today (without shoes)?

kg: ____

During the past 4 weeks, to what extent were you bothered by each of the following?

Lack of appetite	☐ Not at all	☐ Somewhat	☐ Moderately	☐ Very much	☐ Extremely
Aversion to food	☐ Not at all	☐ Somewhat	☐ Moderately	☐ Very much	☐ Extremely
Feeling of unease	☐ Not at all	☐ Somewhat	☐ Moderately	☐ Very much	☐ Extremely
Nausea	☐ Not at all	☐ Somewhat	☐ Moderately	☐ Very much	☐ Extremely
Vomiting	☐ Not at all	☐ Somewhat	☐ Moderately	☐ Very much	☐ Extremely
Itchy skin	☐ Not at all	☐ Somewhat	☐ Moderately	☐ Very much	☐ Extremely
Shortness of breath	☐ Not at all	☐ Somewhat	☐ Moderately	☐ Very much	☐ Extremely
Swollen legs	☐ Not at all	☐ Somewhat	☐ Moderately	☐ Very much	☐ Extremely
Dizziness	☐ Not at all	☐ Somewhat	☐ Moderately	☐ Very much	☐ Extremely
Difficulty remembering	☐ Not at all	☐ Somewhat	☐ Moderately	☐ Very much	☐ Extremely
Difficulty concentrating	☐ Not at all	☐ Somewhat	☐ Moderately	☐ Very much	☐ Extremely

How much of the time during the past 4 weeks did you feel restless legs discomfort?

.	☐ All of the time	☐ Most of the time	☐ A good bit of the time	☐ Some of the time	☐ A little of the time	☐ None of the time

How much of the time during the past 4 weeks did you feel tired?

.	☐ All of the time	☐ Most of the time	☐ A good bit of the time	☐ Some of the time	☐ A little of the time	☐ None of the time

How much bodily pain have you had during the past 4 weeks?

.	☐ None	☐ Very mild	☐ Mild	☐ Moderate	☐ Severe	☐ Very severe

During the past week:

Were you limited in doing regular daily activities?	☐ Not at all	☐ A little	☐ Quite a bit	☐ Very much
Have you been constipated?	☐ Not at all	☐ A little	☐ Quite a bit	☐ Very much
Have you had diarrhoea?	☐ Not at all	☐ A little	☐ Quite a bit	☐ Very much
Have you had to urinate frequently at night?	☐ Not at all	☐ A little	☐ Quite a bit	☐ Very much
Have you had trouble sleeping at night?	☐ Not at all	☐ A little	☐ Quite a bit	☐ Very much
Were you worried about your future health?	☐ Not at all	☐ A little	☐ Quite a bit	☐ Very much

How often do you have difficulty remembering to take all your medication?

.	☐ Daily	☐ Weekly	☐ Monthly	☐ Never/Rarely

General health

In general, would you say your health is:	☐ Excellent	☐ Very good	☐ Good	☐ Fair	☐ Poor
Compared to one year ago, how would you rate your health in general now?	☐ Much better now than one year ago	☐ Somewhat better now than one year ago	☐ About the same as one year ago	☐ Somewhat worse now than one year ago	☐ Much worse now than one year ago

What is most important for you to discuss with your physician in the upcoming consultation? Up to 3 topics may be stated:

Topic 1: ______________________________

Topic 2: ______________________________

Topic 3: ______________________________
