# Supplementary material for: Patient-reported outcome measures for clinical decision-making in outpatient follow-up: validity and reliability of a renal disease questionnaire
Source: J Patient Rep Outcomes. 2021 Oct 16;5:107. doi: 10.1186/s41687-021-00384-0 (PMC8520563; doi:10.1186/s41687-021-00384-0)
Supplement: Supplementary file 4 — Additional file 4. S1. Results from the cognitive interviewing. [file 41687_2021_384_MOESM4_ESM.pdf]

**Supplementary table 1. Results from the cognitive interviewing with patients and clinicians in the outpatient nephrology clinics (n = 14)**	
**Patients, (n = 8)**	**Clinicians, (n = 6)**
**Emerged items**	
Free textbox	Free textbox
Physical pain	Pain
Restless legs	Sexual life
Gastrointestinal function	
**Rephrasing of items**	
Bothered by… instead of plagued by… [In headlines]<br>Worried about your future<br>Health replaces concern for the future<br>Experiencing other problems	Medication adherence<br>Nocturnal urination
**Items removed from the questionnaire**	
Legcramps	Additional question on medication adherence
	Thirst
**Considerations on comprehensiveness and relevance**	
Reflect individual issues<br>Short and concise<br>Use of the questionnaire in the clinical encounter<br>Clear purpose of this questionnaire	Need for teaching patients on the content and purpose of the PRO measures<br>Manageable<br>Meaningful and relevance in the consultation<br>Length and burden to patients
