# Supplementary material for: Patient-reported outcome measures for clinical decision-making in outpatient follow-up: validity and reliability of a renal disease questionnaire
Source: J Patient Rep Outcomes. 2021 Oct 16;5:107. doi: 10.1186/s41687-021-00384-0 (PMC8520563; doi:10.1186/s41687-021-00384-0)
Supplement: Supplementary file 5 — Additional file 5. Agreement and reliability between the items from test 1 to test 2 in original categories (n = 160). [file 41687_2021_384_MOESM5_ESM.pdf]

Supplementary table 2. Agreement and reliability between the items from test 1 to test 2 in original categories (n = 160)								
**Item**	**N**	**Levels**	**%min[1)] (floor)**	**%max [1)] (Ceiling)**	**Agreement/Exp agreement (%)**	**KW (95% CI)**	**Perfect agreement n (%)**	**P-value**
Lack of appetite	159	5	75	2	99.1/89.9	0.91(0.85;0.95)	139(87)	0.36
Aversion to food	159	5	81	2	98.3/92.3	0.85(0.70;0.93)	139(87)	0.007
Nausea	159	4	81	-	98.7/92.0	0.84(0.70;0.92)	144(91)	0.43
Vomiting	158	5	93	-	98.6/95.4	0.69(0.32;0.90)	152(96)	0.42
Itchy skin	158	5	45	1	97.9/88.8	0.81(0.74;0.87)	117(74)	0.001
Dizziness	156	5	54	1	98.9/92.5	0.85(0.78;0.91)	128(82)	0.25
Constipation	159	4	79	-	95.8/85.8	0.70(0.56;0.82)	135(85)	0.41
Diarrhoea	159	4	65	2	97.6/90.5	0.74(0.61;0.85)	130(82)	0.09
Swollen legs	160	4	50	4	97.1/84.7	0.81(0.69;0.88)	129 (81)	0.37
Dyspnoea	159	5	71	-	96.9/87.4	0.75(0.63;0.84)	129(81)	0.14
Daily activities	160	4	60	2	97.6/86.6	0.83(0.74;0.88)	129(81)	0.05
Pain	159	6	50	0.6	97.7/89.6	0.78(0.70;0.86)	112(70)	0.27
Restless legs	159	6	40	4	97.1/86.8	0.78(0.69;0.84)	99(62)	0.27
Fatique	159	6	14	4	97.1/84.7	0.81(0.73;0.87)	88(55)	0.005
Nocturnal awakening	160	4	46	3	96.1/85.0	0.75(0.64;0.84)	120(75)	0.21
Nightly frequent urination	160	4	33	5	96.7/81.8	0.82(0.75;0.88)	121(76)	0.002
Feeling of unease	159	5	63	1	98.2/93.1	0.73(0.63;0.82)	121(76)	0.51
Memory	159	5	46	1	98.4/90.0	0.84(0.77;0.90)	124(78)	0.01
Concentration	158	5	63	0.5	97.4/90.9	0.72(0.53;0.86)	128(81)	0.75
Future concerns	160	4	41	5	96.1/86.3	0.72(0.59;0.82)	118(74)	0.06
Medicine adherence	160	4	89	-	98.3/95.6	0.61(0.34;0.83)	150(93)	0.06
General health	160	5	2	10	97.7/89.9	0.78(0.71;0.83)	105(66)	0.15
Self-rated health compared to last year	160	5	2	2	98.6/94.8	0.73(0.60;0.84)	134(84)	0.69

Abbreviations: Exp: Expected; KW: Weighted kappa with squared weights; CI: Confidence interval; P-value derived from wilcoxon.

[1)]Floor and Ceiling represent the most extreme response category in each item
