# Supplementary material for: Patient-reported outcome measures for clinical decision-making in outpatient follow-up: validity and reliability of a renal disease questionnaire
Source: J Patient Rep Outcomes. 2021 Oct 16;5:107. doi: 10.1186/s41687-021-00384-0 (PMC8520563; doi:10.1186/s41687-021-00384-0)
Supplement: Supplementary file 6 — Additional file 6. Sensitivity analyses showing agreement and reliability between the items from test 1 to test 2 in original categories (n = 204). [file 41687_2021_384_MOESM6_ESM.pdf]

Supplementary table 3. Sensitivity analyses showing agreement and reliability between the items from test 1 to test 2 in original categories (n = 204)						
**Item**	**N**	**Levels**	**Agreement/Exp agreement (%)**	**KW (95% CI)**	**Perfect agreement n (%)**	**P-value**
Lack of appetite	203	5	98.7/90.4	0.87 (0.80;0.92)	174 (87)	0.34
Aversion to food	203	5	98.8/92.9	0.83 (0.72;0.91)	175 (86)	0.01
Nausea	203	4	97.8/91.4	0.76 (0.62;0.85)	173 (85)	0.98
Vomiting	202	5	97.5/94.4	0.56 (0.27;0.82)	174 (86)	0.99
Itchy skin	202	5	97.0/87.5	0.76 (0.65;0.84)	147 (72)	0.01
Dizziness	200	5	98.4/91.7	0.80 (0.70;0.87)	159 (79)	0.19
Constipation	202	4	97.9/92.6	0.72 (0.59;0.80)	168 (83)	0.23
Diarrhoea	202	4	97.0/90.3	0.69 (0.56;0.80)	162 (80)	0.39
Swollen legs	204	4	96.6/84.7	0.78 (0.68;0.85)	155 (75)	0.12
Dyspnoea	202	5	97.8/92.2	0.72 (0.59;0.83)	160 (79)	0.10
Daily activities	204	4	97.2/84.8	0.81 (0.75;0.87)	158 (77)	0.24
Pain	203	6	97.3/88.8	0.76 (0.68;0.84)	139 (68)	0.17
Restless legs	203	6	96.8/85.3	0.78 (0.70;0.84)	123 (60)	0.15
Fatique	203	6	96.9/83.4	0.82 (0.74;0.86)	107 (52)	0.000
Nocturnal awakening	204	5	96.4/85.4	0.75 (0.66;0.83)	156 (76)	0.08
Nightly frequent urination	203	4	96.1/81.7	0.78 (0.69;0.85)	149 (73)	0.01
Feeling of unease	203	5	97.6/92.3	0.69 (0.58;0.78)	151 (74)	0.52
Memory	203	5	98.2/90.1	0.82 (0.75;0.87)	143 (70)	0.03
Concentration	203	5	97.5/91.5	0.72 (0.56;0.84)	145 (71)	0.90
Future concerns	203	5	95.9/83.3	0.75 (0.66;0.84)	151 (74)	0.26
Medicine adherence	204	4	98.1/95.5	0.59 (0.37;0.78)	188 (92)	0.13
Self-rated health	204	5	97.5/88.6	0.78 (0.72;0.83)	134 (65)	0.03
Self-rated health compared to last year	204	5	98.4/94.3	0.72 (0.60;0.81)	167 (81)	0.88
Abbreviations: Exp: Expected; KW: Weighted kappa with squared weights; CI: Confidence interval; P-value derived from wilcoxon.						
