# Supplementary material for: Patient-reported outcome measures for clinical decision-making in outpatient follow-up: validity and reliability of a renal disease questionnaire
Source: J Patient Rep Outcomes. 2021 Oct 16;5:107. doi: 10.1186/s41687-021-00384-0 (PMC8520563; doi:10.1186/s41687-021-00384-0)
Supplement: Supplementary file 7 — Additional file 7. Known group (discriminiative validity) stratified into CKD stage 3b and stage 4 and 5 at time for test 1 among 160 patients with chronic kidney disease. [file 41687_2021_384_MOESM7_ESM.pdf]

Supplementary Table 4. Known groups (discriminative validity) stratified into CKD stage 3b and stage 4 and 5 at time for test 1 among 233 patients with chronic kidney disease				
	**Total**	**Renal function (CKD stages)**		**p-value[1]**
**Item**	**N (%)**	**Stage 3b n = 141 n (%)**	**Stage 4-5 n =92 n (%)**	
**Lack of appetite**				0.03
Not at all	164(70)	108(77)	56(62)	
Somewhat	39(16)	19(14)	20(22)	
Moderately	13(6)	7(5)	6(6)	
Very much	13(6)	5(3)	8(9)	
Extremely	3(2)	2(1)	1(1)	
**Itchy skin**				0.02
Not at all	104 (45)	74 (52)	30 (33)	
Somewhat	82 (36)	41 (29)	41 (45)	
Moderately	21 (9)	14 (10)	7 (8)	
Very much	19 (8)	10 (8)	9 (10)	
Extremely	5 (2)	2 (1)	3 (3)	
**Dyspnoea**				0.85
Not at all	154 (66)	94 (67)	60 (66)	
Somewhat	48 (21)	26 (18)	22 (24)	
Moderately	21 (9)	14 (10)	7 (8)	
Very much	8 (3)	7 (5)	1 (1)	
Extremely	1 (-)	-	1 (1)	
**General health**				<0.001
Excellent	17 (7)	14 (10)	3 (3)	
Very good	49 (21)	36 (26)	13 (14)	
Good	102 (45)	61 (43)	41 (45)	
Fair	52 (22)	27 (19)	25 (28)	
Poor	12 (5)	3 (2)	9 (10)	
**Adherence**				0.45
Daily	-	-	-	
Weekly	7 (3)	5 (4)	2 (2)	
Monthly	14 (6)	6 (4)	8 (9)	
Never/Rarely	210 (91)	129 (92)	81 (89)	
**Dizziness**				0.33
Not at all	115 (50)	74 (52)	41 (46)	
Somewhat	87 (38)	51 (36)	36 (41)	
Moderately	20 (9)	12 (9)	8 (9)	
Very much	5 (2)	2 (2)	3 (3)	
Extremely	3 (1)	2 (1)	1 (1)	
**Concentration**				0.68
Not at all	127 (55)	79 (57)	48 (53)	
Somewhat	72 (31)	41 (29)	31 (34)	
Moderately	25 (11)	16 (11)	9 (10)	
Very much	6 (3)	4 (3)	2 (2)	
Extremely	1 (-)	-	1 (1)	
**Memory**				0.74
Not at all	93 (40)	58 (41)	35 (38)	

Somewhat	96 (41)	56 (40)	40 (44)	
Moderately	30 (14)	22 (15)	8 (9)	
Very much	10 (4)	4 (3)	6 (7)	
Extremely	3 (1)	1 (1)	2 (2)	
**Nausea**				0.09
Not at all	179 (77)	114 (81)	65 (72)	
Somewhat	35 (15)	18 (13)	17 (19)	
Moderately	15 (7)	8 (5)	7 (8)	
Very much	3 (1)	1 (1)	2 (2)	
Extremely	-	-	-	
**Aversion to food**				0.10
Not at all	181 (78)	115 (82)	66 (73)	
Somewhat	29 (13)	15 (11)	14 (15)	
Moderately	13 (6)	7 (5)	6 (6)	
Very much	6 (3)	2 (1)	4 (5)	
Extremely	3 (1)	2 (1)	1 (1)	
**Vomiting**				0.16
Not at all	217 (94)	135 (96)	82 (91)	
Somewhat	10 (4)	3 (2)	7 (8)	
Moderately	4 (2)	3 (2)	1 (1)	
Very much	-	-	-	
Extremely	-	-	-	
**Daily activities**				0.003
Not at all	130 (56)	90 (64)	40 (44)	
A little	67 (29)	34 (24)	33 (36)	
Quite a bit	27 (12)	14 (10)	13 (14)	
Very much	8 (3)	3 (2)	5 (6)	
**Future concerns**				0.04
Not at all	85 (37)	56 (40)	29 (32)	
A little	113 (49)	71 (50)	42 (46)	
Quite a bit	19 (8)	8 (6)	11 (12)	
Very much	15 (6)	6 (4)	9 (10)	
**Nocturnal awakening**				0.77
Not at all	101 (44)	63 (45)	38 (42)	
A little	82 (35)	48 (34)	34 (37)	
Quite a bit	39 (17)	24 (17)	15 (17)	
Very much	10 (4)	6 (4)	4 (4)	
**Fatique**				0.35
All of the time	14 (6)	7 (5)	7 (8)	
Most of the time	31 (13)	16 (11)	15 (16)	
A good bit of the time	45 (19)	28 (20)	17 (19)	
Some of the time	44 (19)	29 (21)	15 (16)	
A little of the time	70 (31)	40 (28)	30 (33)	
None of the time	28 (12)	21 (15)	7 (8)	
**Restless legs**				0.01
All of the time	10 (4)	3 (2)	7 (8)	
Most of the time	14 (6)	8 (6)	6 (7)	
A good bit of the time	23 (10)	14 (10)	9 (19)	
Some of the time	32 (14)	15 (11)	17 (19)	
A little of the time	64 (28)	39 (27)	25 (27)	

None of the time	89 (39)	62 (44)	27 (30)	
**Swollen legs**				<0.001
Not at all	108 (46)	84 (59)	24 (26)	
Somewhat	83 (36)	40 (29)	43 (47)	
Moderately	32 (14)	13 (9)	19 (21)	
Very much	9 (4)	4 (3)	5 (6)	
Extremely	-	-	-	
**Constipation**				0.13
Not at all	173 (75)	111 (78)	62 (69)	
A little	44 (19)	21 (15)	23 (26)	
Quite a bit	13 (6)	8 (6)	5 (5)	
Very much	1 (-)	1 (1)	-	
**Diarrhoea**				0.15
Not at all	154 (67)	99 (71)	55 (61)	
A little	63 (27)	32 (23)	31 (34)	
Quite a bit	6 (4)	6 (4)	4 (4)	
Very much	3 (2)	3 (2)	1 (1)	
**Nightly frequent urination**				0.03
Not at all	69 (30)	50 (36)	19 (21)	
A little	84 (36)	48 (34)	36 (39)	
Quite a bit	62 (27)	34 (24)	28 (31)	
Very much	16 (7)	8 (6)	8 (9)	
**Feeling of unease**				0.13
Not at all	136 (59)	89 (63)	47 (52)	
Somewhat	62 (27)	32 (23)	30 (33)	
Moderately	26 (11)	15 (10)	11 (12)	
Very much	5 (2)	3 (2)	2 (2)	
Extremely	3 (1)	2 (2)	1 (1)	
**Pain**				0.02
None	109 (47)	74 (52)	35 (39)	
Very mild	48 (21)	29 (22)	19 (21)	
Mild	45 (20)	23 (16)	22 (24)	
Moderate	24 (10)	13 (9)	11 (12)	
Severe	3 (1)	2 (1)	1 (1)	
Very severe	3 (1)	-	3 (3)	
**General health compared to last year**				0.02
Much better	8 (3)	7 (5)	1 (1)	
Somewhat better	20 (9)	13 (9)	7 (8)	
About the same	162 (70)	102 (72)	60 (66)	
Somewhat worse	35 (15)	15 (11)	20 (22)	
Much worse	7 (3)	4 (3)	3 (3)	
[1)]p-value testing difference between stage 3b vs. stage 4 and 5. Calculated by Mann-Whitney test				
